# The Effect of Different Doses of Amyloid Beta 1‐42 on Gene Expression Patterns

**DOI:** 10.1002/alz70855_107179

**Published:** 2025-12-24

**Authors:** Zuhal Yurttaş, Tugay Çamoğlu, Ömer Faruk Düzenli, Emrah Yücesan, Erdinc Dursun, Duygu Gezen‐Ak

**Affiliations:** ^1^ Institute of Neurological Sciences, Istanbul University‐Cerrahpaşa, Istanbul, Turkey

## Abstract

**Background:**

Studies have demonstrated that amyloid beta (Aβ) can function as a transcriptional regulator by interacting with the promoter regions of certain genes that play a role in its own production and the development of Alzheimer's Disease(AD) pathology. Moreover, gene expression changes play a crucial role in cellular disorders, particularly during the neurodegeneration process in AD. The aim of this study is to identify genes whose expression changes in response to 0.1µM and 1µM Aβ1‐42 treatments using RNA‐Seq.

**Method:**

RNA isolation was performed from neurally differentiated LUHMES cells following treatment with 0.1 and 1 µM Aβ1‐42 for 48h. Fluorometric and capillary electrophoresis methods were used to assess the quality of the isolated RNA samples. The RNA was then used for cDNA library construction and sequenced on the Illumina NovaSeq 6000 platform. For RNA‐Seq data analysis, the nf‐core/RNAseq v3.17.0 workflow was utilized within the NEXTFLOW v24.10.2 workflow system. Differentially expressed genes (DEGs) were identified using the DESeq2 v1.4.2 package in the RStudio environment. Enrichment analysis of the identified DEGs was conducted using the DAVID tool. Protein‐protein interaction network analysis was performed using the stringApp v2.2.0 and cytoHubba v0.1 tools in the Cytoscape v3.10.13 program.

**Result:**

A total of 125 differentially expressed genes (DEGs) were identified following the 0.1µM Aβ treatment compared to the control group. The top 10 hub genes with the highest interactions were determined as NGF, BMP6, SOX10, THBD, GATA3, IL15, GHR, ACE, ERV3‐1, and ENG. Moreover, among the 1608 DEGs identified in the 1µM Aβ‐treated group compared to the control group, MUS81, TERT, MET, PCG1, FLNA, CALML4, CALML6, CCNL2, SREBF1, and PXDN were found to be common hub genes.

**Conclusion:**

Our findings show that Aβ treatments can alter the expression of numerous genes involved in various pathways, ranging from genes that play a role in intracellular signaling pathways to histone acetylases, in a dose‐dependent manner. Moreover, the results suggest that Aβ may play a role in gene expression changes associated with AD pathogenesis. This study was supported by The Scientific and Technological Research Council of Türkiye(TÜBİTAK) (Project ID: 219Z179) and the Research Fund of Istanbul University‐Cerrahpaşa(Project ID: 37406).

